# Hypotheses relating to the function of the claustrum

**DOI:** 10.3389/fnint.2012.00053

**Published:** 2012-08-02

**Authors:** John Smythies, Lawrence Edelstein, Vilayanur Ramachandran

**Affiliations:** ^1^Center for Brain and Cognition, University of California San Diego, La JollaCA, USA; ^2^Medimark Corporation, Del MarCA, USA

**Keywords:** claustrum, binding, GABAergic interneurons, salience, consciousness, synchrony, oscillations

## Abstract

This paper presents a new hypothesis as to the function of the claustrum. Our basic premise is that the claustrum functions as a detector and integrator of synchrony in the axonal trains in its afferent inputs. In the first place an unexpected stimulus sets up a processed signal to the sensory cortex that initiates a focus of synchronized gamma oscillations therein. This focus may then interact with a general alerting signal conveyed from the reticular formation via cholinergic mechanisms, and with other salient activations set up by the stimulus in other sensory pathways that are relayed to the cortex. This activity is relayed from the cortex to the claustrum, which then processes these several inputs by means of multiple competitive intraclaustral synchronized oscillations at different frequencies. Finally it modulates the synchronized outputs that the claustrum distributes to most cortical and many subcortical structures, including the motor cortex. In this way, during multicenter perceptual and cognitive operations, reverberating claustro-cortical loops potentiate weak intracortical synchronizations by means of connected strong intraclaustral synchronizations. These may also occur without a salient stimulus. By this mechanism, the claustrum may play a strong role in the control of interactive processes in different parts of the brain, and in the control of voluntary behavior. These may include the neural correlates of consciousness. We also consider the role of GABAergic mechanisms and deafferentation plasticity.

## Introduction

In 2005 Crick and Koch suggested that the claustrum might play a key role in information processing in the brain by correlating the separate activity in the different sensory cortices into one coherent activity that “binds” separate sensations into the unitary objects that we experience in consciousness. In a previous paper (Smythies et al., 2012) we outlined an hypothesis as to how this “binding” process might be effected. This hypothesis, however, proved to be unsatisfactory. The present paper presents a new hypothesis that includes not only an account of sensory binding but a number of other functions of the claustrum as well.

The claustrum is broadly divided into three compartments [an anterior-dorsal connected with the somatosensory and motor cortices, a posterior dorsal (visual cortex) and a ventral area (auditory cortex)] (LeVay and Sherk, 1981; Sherk and LeVay, 1981; Sherk, 1986; Edelstein and Denaro, 2004; Crick and Koch, 2005). The claustrum has reciprocal widely distributed anatomical projections to almost all regions of the cortex, as well as to many subcortical structures. A recent high definition diffusion imaging study (Park et al., 2012) reports that the claustrum has connections with the frontal, premotor, ventral anterior cingulate, ventral temporal, visual, motor, somatosensory, olfactory cortices, and most strongly with the entorhinal cortex. It also has connections with some subcortical structures including the putamen, globus pallidus, and lateral amygdala. Until recently, however, the connections between the claustrum and V1 were the subject of some disagreement. Day-Brown et al. (2009) have now published evidence, based on experiments that employed injections of cholera toxin B (CTB), and biotinylated dextran amine tracers, application of antibodies to GABA and glutamic acid decarboxylase, and confocal and electron microscopy, that the two have reciprocal connections. They also noted that four times as many cells were labeled in the claustrum as compared to the dorsal lateral geniculate nucleus following V1 CTB injections.

The auditory cortex may have somewhat different claustral connections than the visual and somatosensory cortices. Clarey and Irvine (1986) report than, in the cat, the input from the primary auditory area to the claustrum is sparse and comes rather from higher auditory areas. Beneyto and Prieto (2001) studied the connections between the claustrum and the auditory cortex by tracer injection methods. They report that all auditory areas have reciprocal connections with the ipsi- and contralateral claustrum. They state, “These findings suggest that the intermediate region of the claustrum integrates inputs from all auditory cortical areas, and then sends the result of such processing back to every auditory cortical field.” By means of differential bidirectional tracer injection experiments in mouse somatosensory cortex Smith et al. (2012) failed to find any significant anatomical projections from the primary somatosensory cortex S1 to the claustrum, whereas they detected dense projections from the same part of the claustrum to both S1 and the motor cortex. They conclude that this shows that the claustrum, in rats, does not function as an integrator of somesthetic and motor function. The experiments of Remedios et al. (2010), however, show that there is a rapid functional connection between the auditory system and the claustrum. Moreover, Zhang et al. (2001), using a similar technique, were able to demonstrate bidirectional connections between the claustrum, on the one hand, and both primary and secondary visual, somatosensory, and auditory cortices on the other.

The claustrum has a well-marked retinotopically organized map of the visual field, as well as an equivalent map of the somatosensory field (Olson and Graybiel, 1980; LeVay and Sherk, 1981; Sherk and LeVay, 1981; Sherk, 1986). It has been claimed that areas of the claustrum in the cat sends “precisely reciprocal” projections back to those area(s) of the cortex whence its inputs derived (LeVay and Sherk, 1981; Sherk and LeVay, 1981; Sherk, 1986). It has also been claimed that the projections to and from the claustrum are diffuse, but quite specific, in both directions (one point to specific points) (Divac et al., 1978; Divac, 1979; Sloniewski et al., 1986). Single cells in the claustrum have been reported to send branched axons to several cortical areas. The same cells receive input from these areas (Rahman and Baizer, 2007). However, there are marked species differences in the anatomy of the claustrum and others claim that the cortico-claustral projection is more diffuse. Crick and Koch summarize their view of the situation thus, “Most regions of the cortex send a projection to the claustrum, usually to many parts of it. Thus, their mappings are far from being a precise local mapping and tend to be somewhat global (that is, all to all, though not completely so).”

The ventral claustrum is also connected to limbic structures, such as the amygdala, subiculum, and cingulate cortex. The claustrum also has a relatively uniform microanatomical structure that would allow what Crick and Koch describe as “widespread intraclaustral interactions.” These, they suggest, may be in the form of waves of information involving dendrodendritic synapses and networks of gap junction linked neurons. They also suggest that claustral neurons “could be especially sensitive to the timing of the inputs.” But they do not suggest any precise mechanism to perform these functions. Rahman and Baizer (2007) also suggest that the claustrum mediates “integration across compartments mediated by inhibitory interneurons (INNs).” On the afferent side, previous reports stated that the majority (75%) of claustral neurons are multisensory in that they respond to stimuli in more than one sensory modality, whereas 25% are unimodal (Spector et al., 1969, 1970). The injection of three different fluorescent tracers into the occipital, frontal, and cingulate cortices results in double- and triple-labeled cells in the claustrum (Li et al., 1986). In contrast, a recent investigation in awake primates found that the great majority of claustral neurons are unimodal (Remedios et al., 2010). On the efferent side, using similar techniques Minciacchi et al. (1985) reported that single claustral neurons project to both anterior and posterior cortex, some ipsilaterally and some contralaterally. This suggests that single claustral cells project to more than one major cortical area. We will return to this point later.

This paper considers what these “waves of information” might be, and what type of timing-related, or other, computations and integrations they might perform. Neurocomputations may take various forms, for example, those based on fixed neural networks (Churchland and Sejnowski, 1999), or non-linear dynamics (Freeman, 2011), or synchronized oscillations (Uhlhaas et al., 2009). In this paper we will focus on the last. One task the claustrum performs may be to provide the cortex with information that allows the cortex to “bind” certain of its on-going activities. In addition the claustrum may not involved solely in sensory “binding”, but it may also be concerned with synchrony detection and modulation in connection with salience processing and a wide range of cognitive processes.

## Our hypothesis

Our basic hypothesis is that the claustrum functions as a synchrony detector, and modulator and integrator of synchronized oscillations (for background see König et al., 1996; Uhlhaas et al., 2009). By “synchrony detector” we mean nothing more elaborate than is implied in the application of two well established principles (1) that neurons respond more robustly to synchronized bursts then to random spikes, and (2) that neurons tend to reproduce in their efferent outputs the pattern of synchronization contained in their afferent inputs. No elaborate timing devices, such as those found in the aural mechanisms used to compute the location of auditory stimuli (Jeffress, 1948; Grothe, 2003), are implied.

When an incoming sensory volley reaches the brain, there are two immediate tasks the brain must perform. The first (1) is to determine whether the information contained in the volley matches the brain's expectation of what that information should be. The second (2) is to find out if the stimulus signals a state of affairs that is potentially threatening or rewarding. The mechanism for (1) entails that, early in the afferent inflow, the incoming down-up messages are matched against the up-down messages of what the brain expects the message to be. Grossberg and Versace (2008) have developed a detailed model (SMART) that locates the matching site in a complex net that involves the LGN and midline non-specific thalamic nuclei. In their model (hypothesis A) a match results in increased synchrony in cerebral gamma oscillations that promotes spike-timing-dependent plasticity (STDP), and is related to the continued use by the brain of the software it is currently using. If a mismatch occurs, the LGN activates the midline and intralaminar nuclei of the thalamus (MILN). This sets in motion a process, which promotes beta synchronization, inhibits STDP and generates modulation of software operations, such as giving “reset” and hypothesis testing instructions. They do not, however, present experimental data that specifically supports this hypothesis. Moreover, the hypothesis presents the following problem. To signal a mismatch the matching mechanism could either send continual matching signals that were interrupted when a mismatch occurred (hypothesis A): or it could remain quiescent until a mismatch occurred, and then send the signal (hypothesis B). The former is extravagant with computing time, whereas the latter is thrifty. Moreover, in human EEG experiments, Kaiser (2003) reports that a bottom-up-driven auditory spatial “mismatch” detection elicits gamma-band activity over the posterior parietal cortex, whereas an auditory pattern “mismatch” report leads to gamma-band enhancements over the anterior temporal and inferior frontal regions. For these reasons hypothesis B might be preferable in the present state of knowledge.

**Figure 1:**
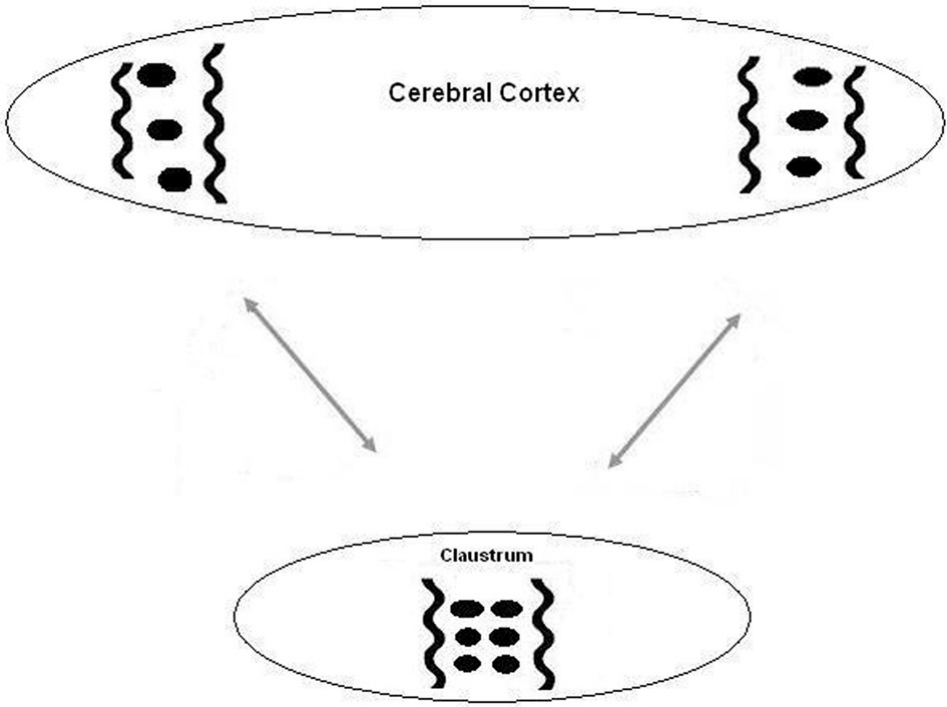
**A diagram of the suggested relation between the cortex and the claustrum.** Oscillations between two distant cortical areas are synchronized by long corticocortical projections. This synchronization is augmented and developed by intraclaustral connections.

So we will first examine the situation where the claustrum is involved with gamma synchronization in response to a mismatch (hypothesis B). The matching site could be a retinotopically identified location in the LGN. In which case, on detecting a mismatch, this would send a signal of axonal spikes, synchronized at a gamma frequency, to the equivalent retinotopic site on the visual map in the claustrum. This relays it promptly to the appropriate part X of the visual cortex where it sets up a short-lived excitation. The claustrum (at least in the tree shrew) projects four times as many fibers to V1 than does the LGN (Day-Brown et al., 2009). With an ever-changing retinal input, such small groups of synchronized cells in the cortex may continually form and reform in competition. Their fate depends on the degree of subsequent attention, recruitment and reinforcement dictated by the nature and details of the stimuli and of the task being performed. The “mismatch” signal from the claustrum may then potentiate activity in one such group at retinotopic location X. An unexpected stimulus also activates the reticular activating system with its widespread projection to the cortex from the nucleus basalis of Meynert (Smythies, 1997, 1999). This system has no map and so cannot signal where X is located. The claustrum may supply this information. The claustral efferent activity might locally augment the cholinergic activation at X. The oscillations of these activated neurons might be synchronized at the gamma frequency of the signal from the claustrum. This synchronization would then be transmitted, via cortico-claustral loops, to the packed densely connected cell bodies (P cells and INs; see Figure 1) in the claustrum. We may suppose that these are in continual state of interacting synchronized oscillations at various frequencies in dynamic competition. In a natural environment salient stimuli rarely affect only one sensory channel. So, when the signal from visual X (e.g., “sight of tiger”) reaches the interior of the claustrum, it may be accompanied by an auditory signal (“roar of tiger”) and an olfactory one (“smell of tiger”). These oscillatory activities may then interact in the internal P cell/IN syncytium, and a derived signal is then sent to the motor cortex (“run for it”). We will return to the possible detailed operations of this system later.

We will now look at the alternative hypothesis (A) suggested by Grossberg and Versace (2008). In this gamma synchronization is associated with a “match” signal, and beta synchronization with a “mismatch” signal. Two points seem relevant. The first is that the gamma signal may not just convey the signal “match,” but it may also carry information about what has been matched, in which case a *null* signal would not be sufficient. Secondly, in the Remedios et al. (2010) experiment, the stimulus was a “familiar jungle scene.” Therefore, the animal was expecting it, and the signal recorded by the experiments might have been a “match” signal. To elicit a “mismatch” signal highly salient stimuli such as the picture of a tiger and the roar of a tiger might be more appropriate.

In hypothesis (B), the mechanism of action of the claustrum would be the same as in hypothesis (A), but the “software” would be different. In hypothesis (A), gamma oscillations are involved in *using* default software, whereas, in hypothesis (B) they are involved in *testing* and *changing* the software program. Thus, in hypothesis (A), a “match” signal from the claustrum to the cortex potentiates synchronized oscillations in one of the competing small groups of cells. In hypothesis (B), the “mismatch” signal does this. In both this process may recruit other groups. These oscillations are then potentiated and integrated by intraclaustral oscillations, leading to an executive signal. In contrast, the processes of testing and changing software in hypothesis (A) are affected by beta oscillations.

However, the following reports on the role of attention and other factors in synchronization suggest that the actual mechanism may be more complicated.

1. Attention to a stimulus enhances frequency synchrony in visual areas (Buffalo et al., 2011). In the gamma range (40–60 Hz) this is largely confined to the superficial layers, whereas the deep layers showed maximum coherence at low frequencies (6–16 Hz). In the superficial layers of V2 and V4, gamma synchrony was increased by attention, whereas in the deep layers, alpha synchrony was reduced by attention.
  This indicates different roles for the same synchrony in different locations.
2. Cardin et al. (2009) report that light-driven activation of fast-spiking interneurons at varied frequencies (8–200 Hz) selectively amplifies gamma oscillations. In contrast, such stimulation of pyramidal neurons amplifies only lower frequency oscillations. They reported that the timing of a sensory input relative to a gamma cycle determines both the amplitude and the precision of evoked responses.
  Thus specificity for gamma oscillations may be dependent on the type of neuron involved.
3. Chalk et al. (2010) measured the local field potential (LFP) and V1 spiking activity in monkeys during an attention-demanding detection task. They reported that, if attention was directed at a visual stimulus in the receptive field of the recorded neurons, decreased LFP gamma power and gamma synchrony resulted. The authors suggested that this decrease could be the result of an attention-mediated reduction of surround inhibition. They further suggested that modulation of synchrony in V1 could be a byproduct of reduced inhibitory drive, rather than a mechanism that directly involved perceptual processing.
  This report suggests a different mechanism altogether for gamma oscillations.
  The next nine reports add yet more complexity.
4. Dockstader et al. (2009) used time-frequency analyses of human somatosensory oscillations with neuromagnetic recordings during stimulation of the median nerve in healthy adults. They reported that selective attention modulated somatosensory oscillations in the alpha, beta, and gamma bands. These were either phase-locked or non-phase-locked to the stimulus. In the primary somatosensory cortex, if the subject's attention was toward the somatosensory stimulus, this resulted in increased gamma band power (30–55 Hz) that was phase-locked to stimulus onset. Such attention also produced an initial desynchronization, followed by increased synchronization, in the beta range, that was not phase-locked to the stimulus.
5. Tallon-Baudry (2009) suggests that “… gamma oscillations are not related to a single cognitive function, and are probably better understood in terms of a population mechanism taking advantage of the neuron's fine temporal tuning: the 10–30 ms time precision imposed by gamma-band rhythms could favor the selective transmission of synchronized information (attention) and foster synaptic plasticity (memory).”
6. Palva and Palva (2007) propose that simultaneous alpha-, beta- (14–30 Hz) and gamma- (30–70 Hz) frequency band oscillations are necessary for unified cognitive operations that mediate, the selection and maintenance of neuronal object representations during working memory, perception, and consciousness.
7. In human EEG studies Haenschel et al. (2000) reported that evoked (stimulus-locked) gamma oscillations preceded beta 1 oscillations in response to novel stimuli.
8. In 10 patients who underwent intracranial electrocorticography, somatosensory-related gamma augmentation involving the post- and pre-central gyri evolved into beta and alpha augmentation. This was later followed by beta and alpha attenuation that involved the post- and pre-central gyri (Fukuda et al., 2010).
9. The prediction of a forthcoming stimulus by a warning cue stimulus was associated with an increase in gamma oscillations overlying occipital areas and a decrease in beta oscillations overlying sensorimotor cortex before the stimulus was presented (Kilner et al., 2005).
10. Gregoriou et al. (2009) suggest that long-range excitatory connections onto interneurons determine whether different pyramidal cell “assemblies” can synchronize at gamma frequencies, whereas excitatory connections onto pyramidal cells determine whether such assemblies can synchronize at beta frequencies.
11. Colgin et al. (2009) report that fast and slow gamma synchronizations in the hippocampus CA1 area have differential effects. The former synchronize with fast gamma oscillations in the medial entorhinal cortex (an area that provides information about the animal's current position) whereas the latter synchronize with slow gamma oscillations in the CA3 area of the hippocampus (an area necessary for memory storage of such information).
12. Fujioka et al. (2009) studied beta and gamma band activities in the auditory cortex of humans during musical beat processing. The beta reaction peaked after each tone but showed no response after an omission. The gamma response, in contrast, peaked after both tones and omissions. This suggests that the beta response, in auditory cortex under these circumstances does not signal “mismatch”; and that the gamma response is related to anticipation rather than a “match” signal.

In view of the many unanswered questions these reports raise, we will confine our hypothesis to limited terms with respect to the functions and frequencies of the synchronizations involved. The claustrum may be involved in detecting, integrating, promoting, and directing synchronized oscillations in a wide range of frequencies and in a number of functional categories. The details await further experimental investigations.

Claustral activation may not occur only in response to mismatch signals. Take, for example, a condition where the subject is engaged in a complex task that involves coordination between sight and touch. Frequent activations of selected visual and tactual cortical neurons will result. On the principle that cells that work together oscillate together, the activated cells will start to synchronize their oscillations. Both sets of cells will spontaneously start synchronized oscillations and will generate axon spikes that will have a timing determined by these oscillations. Each will initially oscillate at its own preferred frequency. This concomitant activity might tend to bind their frequencies of oscillation, and spike timing patterns, into line. These cortical neurons project to the claustrum via specific layer 6 pyramidal cells. The axons of these layer 6 cells synapse on the cell bodies and dendrites of claustral pyramidal cells and will induce in them oscillations at the same frequency.

Evidence that this could occur is based on the following reports. Axons can carry multiple codes in the spatio-temporal patterns of their spike trains (Kayser et al., 2009; Uhlhaas et al., 2009). Codes based on temporal spike-train patterns and spatial populations can nest additional information (codes) based on the relative phase of slow ongoing rhythms at which these (temporal or population) responses occur. Information carried by spike trains can cross synapses and transmit to the post-synaptic neuron. Kumar et al. (2010) state, “The brain is a highly modular structure. To exploit modularity, it is necessary that spiking activity can propagate from one module to another while preserving the information it carries.” Masuda and Aihara (2002) put it thus, “If a real brain uses spike timing as a means of information processing, other neurons receiving spatiotemporal spikes from such sensory neurons must also be capable of treating information included in deterministic interspike intervals.” Asai et al. (2008) found that cortical regular-spiking neurons can propagate filtered temporal information in a reliable way through the thalamo-cortical network, and with high temporal accuracy. This evidence supports the concept that a synchronized afferent input to the claustral cells can cause them to fire at the same frequency and allow them to form reverberating cortico-claustral-cortico loops.

The layer 6 cells in the cortex in the two groups (hearing and vision) are far apart, and are directly connected only by corticocortical axons. The claustral cell bodies connected to them are close together and are connected to each other by short axon collaterals. They are also embedded in a GABAergic syncytium and are linked to each other via the GABAergic gap junction-linked syncytium. This allows very fast communication. It may allow rapid movements of electrolytes and current between them. Electrical synapses between interneurons have been reported to contribute to synchronized firing and network oscillations in the brain (Vervaeke et al., 2010). In the bushy cells of the cochlear nucleus Chanda and Xu-Friedman (2010) have shown that the activation of GABAergic receptors adjust the function of these cells by suppressing the relaying of individual inputs and requiring the coincident activity of multiple inputs. Activation of such GABAergic systems strongly promotes gamma synchronization. So co-temporal activity in the auditory and visual cortex, in this case, may result in a common frequency of oscillation throughout this simple net under the influence of possibly weak cortico-cortical interactions but strong intraclaustral interactions. This particular link up may persist while this particular audio-visual task is being conducted. When the task is completed, the conjoined activity in the cortico-claustro-cortical system would die down, and will be replaced when a new task is undertaken, when the whole process will be repeated again elsewhere.

Thus, this model allows the claustrum to promote synchronized intermodal gamma oscillations in widely separated parts of the cortex. In our previous hypothesis (Smythies et al., 2012) it was thought that to do this the claustrum needed direct inputs to individual claustral cells from axons derived from the two disparate cortices. The data recently obtained by Remedios et al. (2010) suggest that these direct connections do not exist. However, in our present model, such direct connections are not necessary, because the majority of the “binding” is done inside the claustrum. Furthermore, no complex spike codes are involved as in the previous model (Smythies et al., 2012). In non-sensory “higher” cognitive processing operations, that require interaction between any higher cortical areas, the same mechanism may be involved. Weak cortico-cortical promotion of a common oscillation frequency is promoted by a strong intraclaustral promotion. The P cells in the claustrum, embedded in a GABAergic syncytium, may generate a complex, fluctuating, competitive, and dynamic domain of shared and disparate oscillations. This is modulated by the ever-changing pattern of afferent spikes from the cortex, as well as by chemical neuromodulators such as dopamine and input from subcortical structures. For example, positive reward leads to a widespread release of dopamine in the brain. Dopamine promotes gamma synchrony. Remedios et al. (2010) suggest that the short, sharp initial claustral signal from the sense organ to the cortex involves salience and does not involve “binding.” We suggest the hypothesis that this short, sharp initial signal only switches on the cortico-claustral mechanism that does, if circumstances are favorable, effect “binding.”

There is considerable debate on whether the projections of the cortex onto claustral neurons are point-to-point or diffuse. There are also considerable interspecies differences in the anatomy of this system. In the cat this projection is described as “precisely reciprocal” (Sherk, 1986). Little is known about this anatomy in the primate: there are indications that this projection may be more diffuse (Sherk, 1986). However, in this context, it is important to note that the only requirement of our hypothesis is that the activated claustral P cells should receive their input from the cortex via local groups of neurons. This ensures that there is no ambiguity when they fire. Their efferent targets could be back to these neurons by direct return axons, or to other cortical neurons by branched axons. The projections of groups of single cortical neurons to the claustrum may follow a bell-shaped curve. That is, there are more connections with the closest claustral neurons, and less with more distal ones: the more distant the fewer the connections. Then the projection as a whole will consist of a series of partly reciprocal return projections with a degree of overlap. Each small group of claustral neurons may project back to the small group of cortical neurons that gave rise to the axons that connect the two groups in the statistical manner just described. More widespread distribution of claustral impulses may also be mediated by the intraclaustral integrative system of multiple interacting synchronized oscillations that we have described.

### Further data on the role of the claustrum in synchronized oscillations

In a magnetoencephalographic study, Emrich et al. (2006) examined the effect on synchronous oscillations in the brain associated with object perception (shape-from-motion) in six normal volunteers. The stimulus was a computer generated “object” seen against background noise. Each lasted for 4 s. During the MOVE epoch, the object and background moved in counter phase, followed by one of two stationary epochs. In the first—STOP—the motion stopped, and the object remained in the display along with the stationary background noise. In the second—VANISH—the motion stopped and the object was removed from the display, leaving only the stationary background noise. During the MOVE epoch synchronized gamma oscillations (35–45) appeared in the right insula, right claustrum, right superior temporal gyrus (RSTG), and right parahippocampal gyrus (RHG). During the STOP epoch the gamma synchronization remained but changed to a higher frequency (55–72 Hz). The authors propose that claustral activity during the MOVE epoch may be related to conscious awareness of the object, and/or motion cues, and/or memory processes: and that claustral activity during the STOP epoch might be related to awareness of the object only (since the object is not moving).

However, it could be objected that the absence of movement of a previously moving object carries information just as much as the presence of movement does, and both are recorded in memory processes. Therefore, this experiment may actually be relevant to the binding of form and movement, rather than, or in addition to, the “conscious awareness” of an object. Furthermore, in their analysis, the action of the claustrum during the MOVE epoch involves binding of form and movement, but, during the STOP epoch, there is no binding, since the only input to the claustrum, in their analysis, involves form only.

Using an fMRI technique (that provides evidence of activation but not synchronization) Kavounoudias et al. (2008) demonstrated that, in the case of integrating intramodal proprioceptive and tactile information, which resulted in kinesthetic illusions of a clockwise rotation of the right hand, activation of the superior temporal gyrus, the inferior parietal lobe and the claustrum resulted. They suggest that this involved detection of spatial coherence by the inferior parietal lobule and detection of temporal coincidence by the insula structure “usually linked to the relative synchrony of different stimuli.” In an fMRI investigation of color synesthesia activation of the color area V4 resulted (Nunn et al., 2002).

How does our hypothesis explain these findings? Firstly we can note that Emrich et al. (2006) found that the effect they report does not involve the whole cortex, but only the RSTG and the RHG. This suggests that these two loci have a special importance for object recognition of the type examined in this experiment. The STG contains both auditory and polysensory areas (Cappe and Barone, 2005; Smiley and Falchier, 2009). In particular, it contains visual neurons sensitive to both form and the direction of movement (Oram and Perrett, 1996) and projections to the frontal eye fields (Scalaidhe et al., 1997). The RHG contains a higher visual area that processes topographic scenes. The insula has multiple involvements in interoceptive awareness, emotions, and salience. We suggest that the claustrum is involved in tying this inter-center activity together by synchronizing the oscillations in their respective neuronal populations.

Cappe et al. (2012) have suggested a similar mechanism whereby the thalamus, rather than the claustrum acted an integrator of synchronized oscillations. They say, “Some restricted thalamic territories send divergent projections to cortical areas and thus could afford different sensory and/or motor inputs which can be mixed simultaneously. This could support a temporal coincidence mechanism as a synchronizer between remote cortical areas, allowing a higher perceptual saliency of multimodal stimuli.”

## Clinical evidence for binding

We know from the work of Schilder and others (Schilder, 1942) on the manner in which sight returns following injury to the occipital lobe, that the visual field in consciousness is constructed by a tripartite mechanism. After such an injury, the first aspect of sight to recover is movement perception. In this the subject sees pure motion, usually rotary, without any shape or color. Then “space” or “film” colors appear floating about in visual space unattached to any objects. Next, the subjects starts seeing parts of objects, say the handle of a teacup. Lastly these parts join up to form complete objects into which the space colors enter. So activity in the visual field in consciousness can be driven solely by one of these parameters. Their binding into one complete colored, moving object comes later as the cortical areas concerned recover from the injury.

The involvement of the claustrum in perception may involve other processes besides, or even instead of, “binding.” The three different visual parameters—color, shape, and movement—are initially processed by separate cortical mechanisms. Feed-forward over-lapping projections from these areas to the higher visual cortex synapse on single neurons. These can thus react to stimuli from all three lower centers. One neuron may receive inputs from say a lower “red” reactive cell, a “round” receptive cell, and a “moving to the left” sensitive cell. So it has the necessary information for “binding” this input into “a red, round object is moving left.” However, these cells have progressively wider receptive fields the higher in the cortex one goes. Thus these nets may possess the necessary information for binding, but they have poor, or no, information as to where in the visual field these bindings are located. Perhaps this information is supplied by the claustrum, whose projection to the visual cortex is retinotopically ordered. So perhaps the claustral projections to layer IV neurons in the higher visual cortex might provide a type of retinotopically ordered “template” to which the multidomain cells in the higher visual cortex can “refer” in some way. This “referral” might be mediated in some way by interaction of some sort between the binding information carried by each system. The claustral input to the cortex will reactivate the neurons that started the cycle, so the re-entry cycle will continue. These cycles may form the NCCs of consciousness and presumably activate the decision-making centers in the brain so that the appropriate behavior results. We will take up this matter again later.

## The relevance of information from cortical deafferentation experiments

It used to be thought that the neural correlate of a conscious event was activity in a neuron in a specific place in the brain. For example, in the case of vision the place was some location in the defined optic cortex. We now know that the modality of a given sensory neuron is determined, not by where it is, but where its afferent neurons originate. For example, in experiments in blind subjects skilled in Braille, Ptito et al. (2008) showed that magnetic transcranial stimulation of neurons in the optic cortex can result in a somatosensory, and not a visual, experience in these subjects. In these cases, neurons in somatosensory cortex take over the deafferented “visual” cells, and the latter start to process somatic information instead. This activity results in somatosensory sensations in consciousness (sensations that the fingers are being touched) instead of the normal visual sensations. These authors conclude “Our data show that the qualitative character of the subject's experience is not determined by the area of cortex that is active (cortical dominance), but by the source of input to it (cortical deference).”

At a functional level a deafferented cortex (e.g., the visual cortex in the blind) can take over functions of another sensory modality (e.g., hearing) in a number of ways, as reported by Lomber et al. (2010), and Heron et al. (2012). If the sensory inflow of one system is diverted to the cortex of another system (e.g., retinal axons to the auditory thalamus) shortly after birth, this can result in profound changes on different components of cortical circuitry, both at the anatomical and functional levels (Roe et al., 1992; Gao and Pallas, 1999; Pallas et al., 1999; Sharma et al., 2000; Linden and Schreiner, 2003; Chowdhury and DeAngelis, 2008). These changes must be a function of some signaling system in the afferent axons. The nature of this system is not currently known.

The efferent outflow to the claustrum from that part of the deafferented visual cortex, that now performs somatosensory processing (as in the case of the blind subjects skilled in Braille), must enter the claustrum via its medial optic compartment. Here, in spite of being in the “optic” pathway, it is “recognized,” by the claustrum as carrying, in this case, somatosensory information. How could the claustrum do this? There seem to be two possible answers. The first answer is that the claustrum has a mechanism to recognize the unknown modular specific code carried by the afferent axon. The second answer is simpler. The claustral efferents, or at least some of them, return to the same neuron (or neuronal group) that gave rise to the afferent axon. This neuron (or group) is now processing somatosensory information and belongs to the somatosensory system. So the sensation in consciousness that results from this neural cortico-claustral cycling will be a touch on the face, and not a visual sensation. We mentioned earlier that our hypothesis requires only that the efferent outflow from a claustral P cell (or a small group of functionally related P cells) should project in part to the cortical neurons that activated those claustral cells. This is clearly what is happening in the case of the blind subjects skilled in Braille.

We will now return to the question of how the claustrum and the cortex might exchange information. How is this signaling organized and what information might these signals carry? We have suggested that binding is effected mainly by intraclaustral synchrony alignments. The data from cortical deafferentation experiments that we have presented suggests other factors that need to be considered. We brought forward evidence to show that the modality of a neuron is determined by the modality of its afferent axon. The most likely vehicle that could carry such a modality code is the spatio-temporal pattern of spikes in the afferent axon. The only study in this area that we have been able to find reports differences in the character of the spike trains in three different cortical areas “… neuronal spiking patterns are regular in motor areas, random in the visual areas, and bursts in the prefrontal area” (Shinomoto et al., 2009). However, these modality codes would primarily affect the synchrony pattern in their own domains. It might not affect the fact that these individual synchrony bind into one overall pattern. Further data in this area is urgently required. In particular information is needed if a claustral P cell responds to only one visual submode (e.g., color), or whether an individual cell responds to any visual submode (i.e., color, shape, and movement). The mechanism by which modal and sub-modal codes produce the reported major effects in the post-synaptic neurons that they impinge on is currently unknown. However, it may be noteworthy that electrical fields produced by the synchronized oscillation of microtubules play a role in morphogenesis during mitosis and meiosis (kučera and Havelka, 2012; Zhao and Zhan, 2012). This involves alterations in the cell's microstructure. The former cite “in the press” reports of resonant interactions of microtubules with external oscillating electric fields. Therefore it is possible that the electrical fields produced by synchronized oscillations in afferent nerves might induce the structural changes listed above in the postsynaptic neuron by a similar mechanism. Alternatively, or additionally, these changes could be induced by second messenger signaling systems unique to each sensory modality.

### Two binding systems?

We now know that, not only higher cortex, but also probably most of the primary sensory cortex, is polysensory (Falchier et al., 2002; Rockland and Ojima, 2003; Clavagnier et al., 2004; Budinger et al., 2006; Driver and Noesselt, 2008; Ptito et al., 2008; Collignon et al., 2009). Ghazanfar and Schroeder (2006) conclude that, “The nature of most, possibly all, of the neocortex forces us to abandon the notion that the senses ever operate independently during real-world cognition.” Thus, the computations necessary for binding might be all done “in house” within the cortex, in which case we need to explain the need for a second claustral binding mechanism. To approach this question we will first consider the visual system. There is evidence that the claustrum may have two different operative visual systems. The “lower” visual cortex (LVC) (i.e., those areas that receive direct projections from the lateral geniculate body) connects with the dorso-lateral claustrum, whereas those “higher” visual areas that lack a direct input from the lateral geniculate body connect with the ventral claustrum (Sherk, 1986). These “higher” visual areas include areas 20a, 21a, 21b, and the PMLS and PLLS areas of the suprasylvian gyrus, as well as the precuneus (area 7)—a multisensory area with visual and somatosensory inputs. Moreover, the precuneus itself has a topographically organized visual field map, as does its projection to the claustrum. Therefore the claustrum appears to have two visual field maps, one connected to the LVC and the other to some parts of the “higher” visual cortex (HVC) (Sherk, 1986). It is possible that circuits between “unbound” unisensory LVC and the claustrum may have a different function than circuits connecting “bound” polysensory HVC and the claustrum. The former may have more to do with “binding,” and the latter with providing the information where particular objects are located. In polysensory cortex the “binding” would be performed by multi-modal feed-forward nerve nets. In the LVC (neurons with small receptive fields) the sensory data is unbound, whereas in the multisensory HVC (neurons with large receptive fields) the sensory data is already bound intracortically by multiple crossing feed-forward connections. So the cortico-claustral circuits in the LVC may co-operate in the binding, whereas, in the HVC case, they may be more concerned with providing information as to the spatial location of the stimuli as described earlier. In general, these same considerations may apply to the difference between primary “unbound” “lower” sensory cortex and “higher” bound sensory cortex.

### The role of GABAergic interneurons

Our hypothesis suggests that the key to the action of the claustrum lies in its densely packed and tightly interconnected neurons—pyramidal cells and GABAergic INs.

In the cortex and the lateral geniculate nucleus GABAergic interneurons have been found to form extensive polysynaptic bidirectional networks linked by electrical junctions (Fukuda et al., 2006). These authors suggest that these networks support “… the precise synchronization of neuronal populations with differing feature preferences thereby providing a temporal frame for the generation of distributed representations.” Such gap junction linked networks can either promote network synchronization, or trigger rapid network desynchronization, depending on the synaptic input (Vervaeke et al., 2010). This network may promote complex oscillatory interactions of the whole system.

The GABAergic system is also complicated by the presence of a number of different types of GABAergic cell. Rahman and Baizer (2007) analyzed the patterns of immunoreactivity to calcium-binding proteins, GAD, serotonin, nNOS, and the glutamate transporter EAAC1 in the cat claustrum. They found multiple neurochemically defined cell types, suggesting multiple classes of projection neurons and interneurons. Each class was found throughout the entire claustrum, in all functionally defined subdivisions. Kowianski et al. (2009) report the following co-localizations of neuropeptides in the interneurons of the claustrum in rat brain:

- neuropeptide Y with calbindin D28k, calretinin, or parvalbumin
- somatostatin with calbindin D28k
- vasoactive intestinal polypeptide with calretinin

A further subdivision of GABAergic interneurons into five types (in the lateral amygdala) using electronic and electrogenetic parameters has been described by Sosulina et al. (2010). If the same, or similar, subdivisions are found in the claustrum, this might offer scope for further neurocomputational mechanisms. This detailed arrangement suggested to Rahman and Baizer (2007) that many claustral neurons make extensive inter cell type and intraclaustral connections. These connections between the GABAergic network and the INs in the CDNN may serve to allow many modulatory functions as to the fine tuning of the CDNN by neuromodulators, such as norepinephrine and others (Doucette et al., 2001).

In a combined light- and electron-microscopic study Hinova-Palova et al. (2007) showed that the calcium-binding protein parvalbumin is distributed widely and evenly across the cat's claustrum. It occurred in dendritic spines and both spiny and aspiny dendrites. It was also found in boutons at both excitatory (asymmetric) and inhibitory (symmetric) synapses. The authors noted the lack of intrinsic, and possibly functional, heterogeneity, as evidenced by the uniform distribution of PV throughout the cat claustrum. This suggested that the influence of the claustrum on diverse multisensory mechanisms may have more to do with its afferent than efferent relationships. It also indicated its importance in the sensory hierarchy.

In mouse frontal cortex Fino and Yuste (2011) report dense connectivity of GABAergic somatostatin-positive INs. They found that every IN was connected to every pyramidal cell within the range of its axonal tree. They say, “In fact, the complete connectivity that we observe appears in some cases deterministic, as if the circuit has been built to ensure that every interneuron is connected to every single local PC cell … in this way neighboring neurons would have overlapping but not identical connectivity patterns.” It has yet to be determined if this applies to the claustrum.

The cortico-claustral-cortico system may be an example of a strong feed forward inhibitory circuit (FFI) (Bruno, 2011). An FFI is composed of a group of pre-synaptic neurons that directly excite both glutamatergic excitatory (P cells) and GABAergic INNs, and provide greater synaptic input to the latter. The post-synaptic neurons are interconnected. Circuits that lack inhibition simply relay pre-synaptic activity to post-synaptic neurons. In contrast, post-synaptic neurons in an FFI are highly sensitive to the relative timing of action potentials, and this the synchrony, transmitted by the pre-synaptic neurons (Bruno, 2011). Neuromodulators and feedback connections may modulate the temporal sensitivity of such circuits and gate the propagation of synchrony into other layers and cortical areas. The prevalence of strong FFI throughout the central nervous system suggests that synchrony codes and timing-sensitive circuits may be widespread, occurring well beyond sensory thalamus and cortex (Bruno, 2011). However, the evidence is currently lacking as to whether cortico-claustral axons provide the needed greater synaptic input to the inhibitory neurons than to the excitatory neurons needed for a strong FFI system.

## Kappa opioid receptors

An interesting clue may be provided by recent findings concerning the psychoactive drug salvinorin A. This is a specific agonist at kappa opioid receptors. Psychologically it induces an intense sensory synesthesia in which subjects claim that they see sounds and hear sights (Babu et al., 2008; and see Hubbard and Ramachandran, 2005 and Baron-Cohen, 2008). This may be interpreted as an inhibition of sensory binding. In our present context it is interesting that activation of the kappa opioid receptor has been shown to inhibit the release of GABA in the bed nucleus of the stria terminalis by a pre-synaptic mechanism (Li et al., 2012a). If the same holds in the claustrum, this would provide direct evidence that the GABAergic system in the claustrum may be related to sensory binding. The claustrum contains particularly high levels of mRNA for the kappa receptor (Meng et al., 1993; Mansour et al., 1994). The binding of kappa-1 opioid-stimulated [35S] GTPgammaS (a marker of the kappa opioid receptor) is also particularly high in the ventral claustrum (Sim-Selley et al., 1999).

## The relevance of illusory conjunctions and attention

Attention plays a prominent role in binding. Vohn et al. (2007) have shown that the right claustrum is involved both in within-modal and cross-modal (auditory and visual) divided attention performance as part of a circuit that includes the prefrontal cortex and the inferior parietal cortex. Also relevant to the binding problem is the question of “illusory conjunctions” (Treisman and Schmidt, 1982; Crick and Koch, 2003). If separate features of an object (e.g., “red square”/“moving left”) are processed in different brain areas (i.e., the color area V4 and the motion area MT), which results in the loss of their topographic location label, and if two objects are simultaneously presented—e.g., a red one moving left and a green one moving right, then how does the brain compute which color goes with which motion? One possibility is that Crick's “searchlight” of attention is directed toward different portions of the visual scene at an early stage of processing when topographical information is still present (e.g., area 17 of the primary visual cortex, or the claustrum). If the spotlight permits “red” and “left” to go through, then they will be bound. This explains why, if a red triangle and green square are briefly presented and masked, subjects see illusory conjunctions in 50% of trials. This is because there has been enough time to process the features separately, but not enough time for (in our scheme) claustro-striate iterations to ensure correct binding through attention. Crick and Treisman suggested that that the nucleus reticularis thalami is involved. We would argue, from the current evidence, that ascending brainstem efferents to the claustrum interacting iteratively with topographically organized area 17 may constitute the searchlight (see also Smythies, 1997).

Some difference in the motor and sensory functions of the claustrum may be suggested by the observations that the claustral neurons projecting to the contralateral motor cortex are predominantly pyramidal in shape, whereas the predominant claustral neurons projecting to the somatosensory, visual, and auditory cortices are mainly oval on shape (Sadowski et al., 1997).

Naghavi et al. (2007) have reported that, whereas the claustum/insula area (CIA) was activated by attentionally-focused congruent stimuli (e.g., sight of a cat's face and hearing a meow) it failed to be activated by attentionally-focused non-congruent stimuli from the same location in external space e.g., the visual stimulus of a cat's face with the contemporaneous auditory stimulus of a dog's bark. They suggest that this indicates that the CIA must be involved in the “analysis of the content of stimuli.” However, it must be noted that the CIA did not react at all to the cat's face/dog's bark. So it can hardly be said to have extracted the information “cat” from its visual input, and “dog” from its auditory input, compared the two, computed that they are incongruent and rejected them from its binding operation. Furthermore, we suggest that the CIA simply does not have the computational capacity to decipher all the immense flow of information that passes through it. The decision that “cat” and “bark” do not go together must have been made in the higher cortex before it activates the CIA.

So we would suggest is that the CIA does not react in this experiment to “cat/bark” and “dog/meow” because these improbable signals have been suppressed upstream. This would be an example of the same sort of mechanism that operates in the experiment reported by Kovács et al. (1996). In this experiment they took two photographs, one of a monkey's face and the second of a leafy tropical jungle. They converted these into two pastiches each composed of portions of each photo, so that in the location where one photo showed part of the monkey's face the other showed leafy jungle. Then each pastiche was shown separately to each retina, so that retinal rivalry occurred. Under these circumstances, the subject did not see what was actually there—that is the two pastiches alternating—but rather a complete monkey face alternating with a complete leafy jungle. Clearly the brain had suppressed the improbable mixed pastiche in favor of what it was familiar with (and thus computed to be more probable). Many other experiments, based on stimuli such as moving plaid patterns, have shown this phenomenon, where the perception of an improbable input is suppressed by the brain, and replaced with the perception of what it computes to be a more probable one (see Ramachandran and Anstis, 1983).

## The role of the claustrum in cognitive processing

So far we have focused upon the original Crick-Koch hypothesis that is based on the idea that the claustrum is involved in binding features of the sensory stimulus. We have suggested that a spike burst synchrony detecting, modulating, and transmitting mechanism may be involved. However, it is clear that the claustrum is involved in many other processes besides sensory binding. The claustrum has bidirectional connections with non-sensory limbic, temporal, and frontal cortices, as much as with the sensory-motor cortex. The claustrum also has a massive input from all the major neuromodulator circuits (Lacković et al., 1990; Meador-Woodruff et al., 1991; Sutoo et al., 1994; Baizer, 2001; Edelstein and Denaro, 2004; Gill et al., 2006; Eggan and Lewis, 2007; Das, 2010). The ventral claustrum has extensive connections with limbic areas such as the anterior cingulate gyrus, amygdala, hippocampus, and others. Why would this information, relating largely to emotions, reinforcement, and motivation, be required by a system concerned with sensory binding? One answer might be that the claustrum is composed of synchrony modulators involved in binding between limbic operations (emotions, etc.) and the sensory and motor systems. The dorsal claustrum has connections mainly with sensory and motor cortices, the ventral claustrum has connections mainly with limbic cortex and subcortical structures, and the rostral claustrum mainly with frontal cortex (LeVay and Sherk, 1981; Sherk and LeVay, 1981; Sherk, 1986). The anterior dorsal, posterior dorsal, and part of the ventral subdivisions could be concerned with sensory-motor binding in the manner we described earlier. The anterior and another part of the ventral subdivisions could be concerned with reinforcement and other limbic functions. Thus, the claustrum could cooperate with the sensory cortex for sensory binding, with the limbic system for emotional coordination to allow modulation of behavior by complex patterns of reinforcement, and with the decision-making mechanisms in the prefrontal cortex to coordinate “higher brain” functions (in response in each case to specific inputs from these different loci).

Our hypothesis suggests that, in these cognitive and limbic processes, the role of the claustrum may be put as follows. The claustrum may become activated whenever a computational process involves more than one brain area. If two such areas are involved, for example, the synchronized activities in each are coordinated by the claustrum in the manner we have described—scattered weak intercortical synchronizations are potentiated and processed by strong intraclaustral synchronizations. We can call this “cognitive binding.”

Recent research suggests what some of these “higher brain functions” may be. Volz et al. (2010) carried out fMRI studies of retrieval fluency in normal subjects. This is defined as how long it takes to retrieve a trace from long-term memory. If one of two objects is retrieved more quickly this indicates that that one has a higher value than the other. This measure was accompanied by another that assessed the emotional feeling in the subject of the “rightness” of that memory (a feeling-of-knowing judgment). The authors suggest that a number of brain processes could contribute to these measures including “… the ease with which such memories are bound together.” Their results were that these procedures were associated with activation of the dorsal claustrum, but not the ventromedial prefrontal cortex. The authors suggest that their findings indicate that the claustrum may also bind semantic and emotional information in addition to sensory information. Tian et al. (2011) have investigated another possible function in which the claustrum may play a role—the mental preparation of successful insight problem solving (that is the “Aha” experience as exemplified by Archimedes). Using fMRI they showed that successful preparation coding was associated with activation of a circuit that included the parts of the frontal and temporal cortices, the cerebellum and the bilateral claustrum. Our postulated claustral synchrony detection and augmenting mechanism may be plugged into a number of different neural circuits engaged in different distributed computations. The claustrum is also engaged in the rapid interhemispheric dissemination of information needed for the bilateral coordination of movement regulation (Smith and Alloway, 2010). The mechanism we propose might effect this by “binding” information from two inputs, one from each ipsilateral motor system.

The mechanism described in our hypothesis may underlie the proposed mechanisms of timing and time perception that rely on “beat-frequency” patterns generated by cortical oscillations (see Matell and Meck, 2004; Buhusi and Meck, 2005; Allman and Meck, 2012).

## The saliency detection hypothesis

Remedios et al. (2010) have suggested that the function of the claustrum is related to salience and salience detection. This was based on their own experiments conducted on awake primates. The stimuli were naturalistic video recordings, naturalistic auditory recordings, and both presented together. Recordings were taken from individual neurons in various parts of the claustrum. Their results confirmed the fact that visual neurons and auditory neurons are located in different loci of the claustrum (ventral and dorso-central, respectively). However, they reported that the neurons reacted to visual or auditory stimuli, but not both. They added some other interesting details. In all cases the response latencies were short and the responses were brief and stereotyped—a short strong transient followed by a diminished sustained response. Even the responses of claustral neurons to long naturalistic sounds were in the form of brief transients. This was in contrast to what the same group, using the same stimuli, had found in higher sensory cortex, where considerable fractions of integrating or bimodal neurons were found in the auditory cortex (Kayser et al., 2008) and the superior temporal cortex (Dahl et al., 2010). Furthermore, Remedios et al. (2010) tested for multisensory integration in the claustrum by looking for instances where the response to one stimulus is modulated by a stimulus in a different modality. No such modulations were found. These workers suggested that previous reports of multimodal neurons in the claustrum were probably contaminated by influences from the insula. They concluded that the claustrum is most likely to act as a salience detector. They note that inputs from the early cortices and/or thalamic nuclei are rapidly fed to the claustrum, and are rapidly directed from there widespread to the higher cortex.

We agree with the hypothesis that one function of the claustrum may be saliency detection. However, as we have seen, it is not necessary for Crick and Koch type binding that each claustral cell should to receive axons from both cortical areas whose functions are to be bound. This binding may be effected inside the claustrum in the manner we have suggested. Also, even if the input to claustral cells is unimodal, the output axons from claustral P cells commonly bifurcate and project to more than one modal type of cortex (Divac et al., 1978; Divac, 1979; Sloniewski et al., 1986; Rahman and Baizer, 2007). The experiments reported by Remedios et al. (2010) only produce strong evidence that the input to individual claustral P cells in not intermodal. However, this evidence is not relevant to the possibility of intraclaustral binding operations, nor to the question of whether the output of single claustral P cells projects to more than one cortical modality.

Moreover, in support of the saliency hypothesis, spike-timing (synchrony) codes may carry information in addition to that relating to the properties of the stimulus. Doucette et al. (2001) suggest another account of what information synchronized spikes may be carrying. This leads to a wider concept of what the function of the postulated spike burst synchrony detection mechanism of the claustrum might be. Doucette et al. (2001) studied spike-timing codes in the olfactory system and came up with a challenging result. They report that the number of synchronous spikes (SS) fired by pairs of olfactory bulbar neurons signals, not stimulus properties, but the salient information of whether the odor is associated with reward or not. The SS fall below the spontaneous activity level for unrewarded odors, and rise above it in the case of rewarded odors. The authors suggest that this is an easily understood and implemented population temporal code, the decoding of which simply requires downstream coincidence detectors, connected to decision-making networks, that take input from both members of the neuron pair. Doucette et al. (2001) then found that the SS rate is modulated by noradrenergic input to the bulb. In this way the SS code can signal the reinforcement significance of the stimulus. Katz and Maier (2011) comment in the same issue of Neuron “It is also unclear whether, when coherently firing neurons are studied in larger ensembles, the observable patterns will become more complicated.” Katz and Maier conclude, “Thus, these are important, novel data added to a growing corpus suggesting that “sensory” coding is as much about the stimulus in context as what the stimulus physically is.” That is to say that the reinforcement significance of most stimuli will be affected by other events happening in the environment. For example, the last meal chosen and eaten by a condemned prisoner will not carry the same reinforcement value that it should.

Perhaps, then, the claustrum could be functioning, in some locations, as such a reinforcement related “downstream coincidence detector” on a global scale in larger assemblies? In some areas (such as the anterior and part of the ventral claustrum) the spikes impinging on the coincidence (synchrony) detectors in the claustrum may carry salient information relating to the reinforcement value of the stimulus rather than to the sensory properties of the stimulus. The function of this might be to provide a metastable network that enables the brain to compute the significance of a stimulus in its complex global context. That is, a stimulus can be signaling reward, or the opposite, depending on its contextual environment—on what surrounds it. In other words the brain must have a global mechanism that needs input from other sensory systems to evaluate the reinforcement significance of a single stimulus presented in a complex environment.

It is possible, therefore, that the claustrum might perform both functions described above—computing binding and computing global reinforcement—in different locations. The brain contains several systems involved in salience detection—for example the mesolimbic dopamine system (Enomoto et al., 2011; Friston et al., 2012). Another salience network includes the dorsal anterior cingulate cortex, middle and inferior temporal cortex and the fronto-insular cortex (Cauda et al., 2011; Yuan et al., 2012). Day-Brown et al. (2010) report the existence of a direct projection from the superior colliculus to the amygdala in the tree shrew that they suggest alerts the animal to the presence of danger, i.e., carries negative salience. There is at least one system involved in salience and executive control of which the claustrum is an integral part, and that is the rubral network based on the red nucleus. This consists, in addition to the red nucleus, the cerebellum, mesencephalon, substantia nigra, hypothalamus, pallidum, thalamus, insula, claustrum, posterior hippocampus, precuneus, and occipital, prefrontal, and fronto-opercular cortices (Nioche et al., 2009). Little is known about the functions of this circuit.

It is therefore necessary to distinguish between several types of “salience.” “Salience” can involve the process by which new and possibly important stimuli are identified and evaluated. In this form of salience detection, the direct sensory input from the sense organ is compared with feedback from the higher cortex that predicts what the input should be. It can also mean the process by which information about the reinforcement history of stimuli is processed.

We also need to enquire further how reinforcing and salient stimuli could affect this system. The cortex receives widespread projections from dopamine neurons in the ventral tegmental area (VTA) that are activated when a stimulus with more than expected reward is received. Representations of sensory stimuli in the cerebral cortex can undergo progressive and extensive remodeling according to the behavioral importance of the stimuli (Bao et al., 2001). There are several reports that dopamine promotes neuronal oscillations. At the microcircuit level, during striatal network activity, the selective activation of either D1 or D2 receptors results in an increase of neuronal synchronization (Carrillo-Reid et al., 2011). In bullfrog retinal ganglion cells dopamine promotes synchronization (Li et al., 2012b). Dopaminergic stimulation can either promote, modulate or inhibit oscillatory activity depending on the structure innervated, and the frequency of the oscillation (Lee et al., 2004). For example, Cassidy et al. (2002) studied human patients following surgery for Parkinson's disease. Without medication coherent oscillations were apparent between the subthalamic nucleus and the globus pallidus interna at <30 Hz. After exogenous dopamine stimulation the coherence frequency increased to 70–85 Hz. A neurochemical mechanism for this effect is suggested by Kuznetsova and Deth (2008). They found that dopamine, acting on D4 receptors, promotes synchronization of oscillations in cortical pyramidal cell—interneuron networks. This is mediated by modulation of phospholipid methylation that alters the kinetics of potassium channels. P cell-interneuron networks are prominent in the claustrum. Perhaps the claustrum is involved in this type of salient activity?

For an example from another system, anxiety is reported to be associated with theta-frequency synchronization between the ventral hippocampus and the mPFC (Adhikari et al., 2010). These authors suggest that such synchronization is a general mechanism by which the hippocampus communicates with downstream structures of behavioral relevance. Other structures besides the hippocampus may do the same.

Therefore we fully agree with the conclusion by Remedios et al. (2010) and Remedios (2012) that the claustrum is involved in a variety of higher cognitive processes, including saliency relevant ones.

## Advantages of our hypothesis

The advantages of our hypothesis seem to us to be:

- Its simplicity of action. It does not need any complex mechanisms, such as axonal spike time pattern analysis. The claustrum simply detects synchrony, processes intraclaustral synchronies, and promotes intermodal synchrony in functionally connected cortical and subcortical areas.
- Its low neurocomputational cost. The claustrum is not concerned with the informational content of the spike trains fed into it, only with their degree of synchrony.
- It accounts for how one mechanism can exert functions that affects many “higher” brain functions.
- It gives a key role to the GABAergic interneuron system in claustral function.

If we ask “What makes the claustrum special?” we would reply that it reproduces in a small volume activity from all over the much larger volume of the rest of the brain. This promotes the development of multiple, fluctuating, and differential synchronous oscillations essential to the claustrum's postulated role. No other brain structure has this property.

The differences between our previous hypothesis (H1) and our present hypotheses (H2) are as follows. (1) The essential feature in H1 was a series of pyramidal cells that acted as [AND gates] which detected synchronous axonal spikes, and reported back to the part of the cortex whence these spikes originated. (2) The function of the GABAergic INs was allotted merely to noise reduction. (3) In H1 a bimodal innervation of claustral P cells was required. In H2: (1) the P cells and INs act as units that detect synchronized oscillations in the afferent inflow, and then integrate these by its competitive intraclaustral oscillatory system. The claustrum then projects executive signals based on this integration to the motor cortex and limbic system. (2) Noise reduction has been eliminated. (3) Bimodal innervation is not required.

We feel that we should mention that we have carried an extensive exploration of other possible hypotheses, in relation to the “binding” function of the claustrum, based on other fixed nerve net systems, spike train spatio-temporal codes, and non-linear dynamics, but failed to develop any promising candidates in those fields.

### Problems with the hypothesis?

Duffau et al. (2007) conducted total unilateral excision of the claustrum and insula for glioma in 42 patients. They could not detect any neurological or psychological abnormalities post-operatively in any of the cases, who all returned to their normal professional lives, except for three who suffered a stroke due to vascular complications. The authors concluded that the system must have an effective compensatory system, but do not venture an opinion as to what this might be. We suggest that the answer may lie in the fact that the claustrum has extensive connections with the contralateral cortex, at least in the cat (Sherk, 1986). She states—

“Norita (1977) found that all areas of cortex tested received input from two roughly corresponding regions in the two claustra, though the projection from the contralateral side was always considerably weaker. These findings have been confirmed by others, chiefly Macchi et al. (1981).”

## Experiments to test our hypothesis

Central to our hypothesis is the question of how does a neuron react if its input consists of two trains of spikes produced by two groups of afferent neurons oscillating at different frequencies. Gielen et al. (2010) have conducted a theoretical and computer simulation study, and have come to the conclusion that the target neuron(s) fired in a pattern that was highly phase-locked with the larger of the two afferent signals. The question then arises what is the result if they are of equal size. However, different types of pyramidal cell react in different ways, and there is currently no information as to which type the claustral pyramidal cells belong (Gielen, personal communication). An extended answer to this problem might produce essential evidence as to the possible role of such a mechanism in the claustrum. Interesting basic data on the synchronization properties of pyramidal neurons are provided by Di Garbo et al. (2007). Relevant information might be obtained by experiments that fed axonal bursts synchronized at different frequencies into the claustrum, and recorded what form of synchronized bursts appeared in the relevant outputs from the claustrum.

In particular, the problem to be solved might be put in the following form. The claustrum consists of a fairly uniform tangled array of pyramidal cells and a wide variety of GABAergic INNs, all densely interconnected by axon collaterals of the P cells, axons, and dendrites of the INNs, and the gap junction linked syncytium formed by the INNs. This complex system receives afferent axons from most brain areas. So what is the likely form of interaction between all the different synchronized oscillations, both of intrinsic and extrinsic origin, set up by this system, and how would this affect the output patterns of the P cells?

## Conclusion

This paper presents a specific hypothesis on the mechanism of action of the claustrum. It suggests that the claustrum functions as a synchrony detector that detects and reacts to the degree of synchrony contained in spike bursts in their selective input axons. This synchrony is facilitated by intraclaustral synchronizing interactions between the P cells, and GABAergic IN cells, and is maintained in dynamic cortico-claustral-cortical reverberating circuits. These cycles may function as the neural correlates of consciousness. In the case of the ventral claustrum this system may relate to the reinforcement (salience) value of stimuli and other higher brain functions of the claustrum in some cases. We also discuss a complementary hypothesis related to salience detection and estimation.

Thus our position is very close to the hypothesis proposed by Crick and Koch (2005). They describe “widespread intraclaustral interactions,” that may be in the form of “waves of information {that} can travel within the claustrum.” This may involve, they suggest further, dendrodendritic synapses and networks of gap junction linked neurons. They also suggest that claustral neurons “could be especially sensitive to the timing of the inputs.” We feel that we have merely supplied some details of this process. We also support the hypothesis presented by Remedios et al. (2010) if this is stated that one function of the claustrum is related to salience processing.

In a study of sparse and dense codes in the brain during exposure to language passages and music Lloyd (2011) found that the resulting fMRI activity has properties more similar to music than to language. Crick and Koch (2005) famously likened the claustrum to the conductor of the orchestra. We agree that the evidence points to this role for the claustrum, as the conductor of an orchestra that plays a symphony woven out of a continual shimmering interplay of information-laden harmonies built out of synchronized potentials oscillating at many different frequencies.

## Postscript

### The function of the claustrum in a nutshell: the brain's war room

Any large organization, composed of many modules that process incoming information, and has an output in the form of intelligent behavior, will have a problem with internal information flow. Each module is busy processing its own input and providing an output, but how does one module know what another is doing, and how does the organism as a whole know what is going on, and what best to do next? Intercommunication between the modules soon leads to overload. Direct intermodular connections, however extensive, and non-linear dynamics can only go so far.

For example, in an army, one unit can make judgments about what to do next by observing what other units beside it in the line are doing, and making rational decisions. These may be right in the local context, but wrong in the context of the overall war plan. In the Napoleonic wars this is what generals in charge of brigades had to do during the time it took a galloping horse to get from Paris to Madrid. Another example is the battle of New Orleans, which was fought after the War of 1812 had ended. The advent of wireless telegraphy changed the situation. During WW2 Churchill set up a control room in the basement of 10 Downing Street. This was equipped with a large map in the center of the room, where small models of the planes, ships, tanks, etc. involved could be moved about in response to telegraphed reports from the front. The generals could review the whole situation at a glance, and make instant decisions about what orders to send back.

We suggest that the claustrum, with its own internal maps, is doing the same thing. It not only “binds” sensory information, as Crick and Koch proposed, but it is receives edited incoming information (evaluated for novelty and salience) from five different channels (via cortico-claustral and thalamoclaustral afferents). It then calls in further reports and analyses, when needed, from areas of cortex it activates (by means of a web of synchronized oscillations and axon bursts). Lastly the claustral circuits integrate all this activity (via intraclaustral synchronized oscillations), to come up with a series of executive orders transmitted to the motor cortex. In other words, the claustrum may do for its brain what Churchill's War Room did for the Allied armies.

We do not imply that this model requires the infamous little green man in the War Room reading all this incoming data and making decisions based on it. The evaluation of the data and the decisions are both functions of the competitively interacting rhythms.

## Note added in proof

This note added in proof details how our hypothesis explains the new experimental data reported by Ryan Remedios in his invited lecture entitled “The Claustrum and the Orchestra of Cognitive Control,” presented at the *First Annual Francis Crick Memorial Conference: Consciousness in Human and Non-human Animals*, held at Churchill College, Cambridge on July 7th, 2012 (Remedios, 2012)^1^.

At the Conference (which was live-streamed over the internet), Remedios presented the results of a series of experiments he had conducted in rats using a molecular lesioning technique involving saporin (a ribosome-inactivating protein), to effectively eliminate most if not all of the claustrum. When these “aclaustral” animals were placed on the center platform of an elevated eight-arm radial maze, they did not explore the maze as did the control subjects, but, in the speaker's words, remained “frozen” at its center, with infrequent short forays into one of the arms. Likewise, rats placed in a running situation on a Rotarod continued running far longer than did the control subjects.

Our hypothesis suggests that sensory—and much cognitive—discrimination and integration (leading to an executive order) is effected by competitive synchronous gamma oscillations. This mechanism operates at a weak level within the cortex (via corticocortical synchronizations), and at a stronger level within the claustrum (via intraclaustral synchronizations), the connection between the two maintained by cortico-claustrocortical loops.

In the case of the lesioned rat “stuck” at the center of the maze, we suggest that this has nothing to do with behavioral or emotional “freezing,” but that it is a case of the “donkey caught between two identical bales of hay” syndrome. The loss of the claustrum results in the subject losing its ability to choose between what are very similar arms of the maze. The weak signals, produced by the now isolated cortical system, are insufficient to trigger the executive order necessary for the rat to enter an arm of the maze. In effect, the lesioned animal can no longer make up its mind. Likewise, the running lesioned animal, by the operation of a similar mechanism, cannot decide when to stop.

In addition, with the use of BOLD fMRI in these saporin-lesioned rats, Remedios reported increased widespread positive correlations across the various sensory cortical areas, as well as increased activity in the prefrontal cortex (PFC). Our hypothesis also explains this finding, premised on the well-known principle that, when one mechanism in the brain is inactivated, it immediately tries to activate some other mechanism(s) to compensate for the loss. In this case, the loss of the integrating activity of the claustrum leads to an expected compensatory increase in activity in the corticocortical system. In this regard, we also wish to note that our hypothesis serves to explain the multiple cognitive effects of claustral activity, as detailed in this paper. It is unclear to us how an “interhemispheric bidirectional PFC-claustrum network,” as detailed by Remedios in his lecture (without specifics offered as to what its function might be), would accomplish this.

Lastly, we have already commented in this paper on the point raised by Christof Koch during the Q…A session, which immediately followed the Remedios lecture, referring to the lack of salience in the stimuli used in his monkey claustrum unit physiology study (Remedios et al., 2010), which was also presented during his talk. We agree with his point that, in order to test a salience hypothesis, it is necessary to use salient stimuli, which, in the case of the monkey claustrum study (Remedios et al., 2010), would be a multisensory/multimodal task.

### Conflict of interest statement

The authors declare that the research was conducted in the absence of any commercial or financial relationships that could be construed as a potential conflict of interest.

## Footnotes

^1^http://fcmconference.org/#talks
